# Effects of *Plasmodium berghei* infection on the expression of salivary gland immune-related genes in the *Anopheles stephensi* mosquito

**DOI:** 10.1186/s12936-025-05430-6

**Published:** 2025-06-06

**Authors:** Sakineh Pirahmadi, Zahra Sadat Mousavi Shafi, Zeinab Mohammadi Firouz, Akram Abouie Mehrizi, Jafar J. Sani, Hemn Yousefi, Sepideh Siasi, Abbasali Raz, Flora Forouzesh, Leila Darzi

**Affiliations:** 1 Malaria and Vector Research Group (MVRG), Biotechnology Research Center (BRC), Pasteur Institute of Iran, Tehran, Iran; 2 Department of Genetics, Faculty of Advanced Science and Technology, Tehran Medical Sciences, Islamic Azad University, Tehran, Iran; 3 Department of Medical Biotechnology, Faculty of Medical Sciences, Tarbiat Modares University, Tehran, Iran

**Keywords:** *Anophelesstephensi*, *Plasmodiumberghei*, Salivary glands, Gene expression, Innate immunity

## Abstract

**Background:**

Achieving malaria eradication by 2050 will require the development of novel transmission-blocking strategies alongside existing and emerging control measures. Since the innate immune responses of *Anopheles* salivary glands determine its vectorial capacity, a detailed assessment of vector-parasite interactions could help identify novel targets that play key roles in the immune response against *Plasmodium*. In this study, six candidate immune-related genes from *Anopheles stephensi* salivary gland transcriptomic datasets were selected, and their expression changes were assessed following *Plasmodium berghei* infection.

**Methods:**

Using RT-qPCR, gene expression profiles at 18 days (early phase) and 21 days (late phase) post-infection were analysed, and the results were compared with those of uninfected mosquitoes.

**Results:**

A significant upregulation of *LRIM8A* and *DEF1* gene expression was observed at both time points, whereas *TEP-12* expression was significantly increased only at day 21. However, no significant changes were observed for *P37NB*, *CLIPA4*, and *CLIPC4*. Among the highly expressed genes, *LRIM8A* exhibited the highest expression during both the early and later phases of salivary gland infection.

**Conclusions:**

The highest expression levels of *LRIM8A* at both early and late phases of salivary gland infection underscore its potential as a key immune effector. However, further functional assays are required to validate the role of *LRIM8A* in mosquito innate immunity. A deeper understanding of the immune mechanisms in *Anopheles* following *Plasmodium* infection could contribute to the development of novel malaria control strategies.

**Graphical Abstract:**

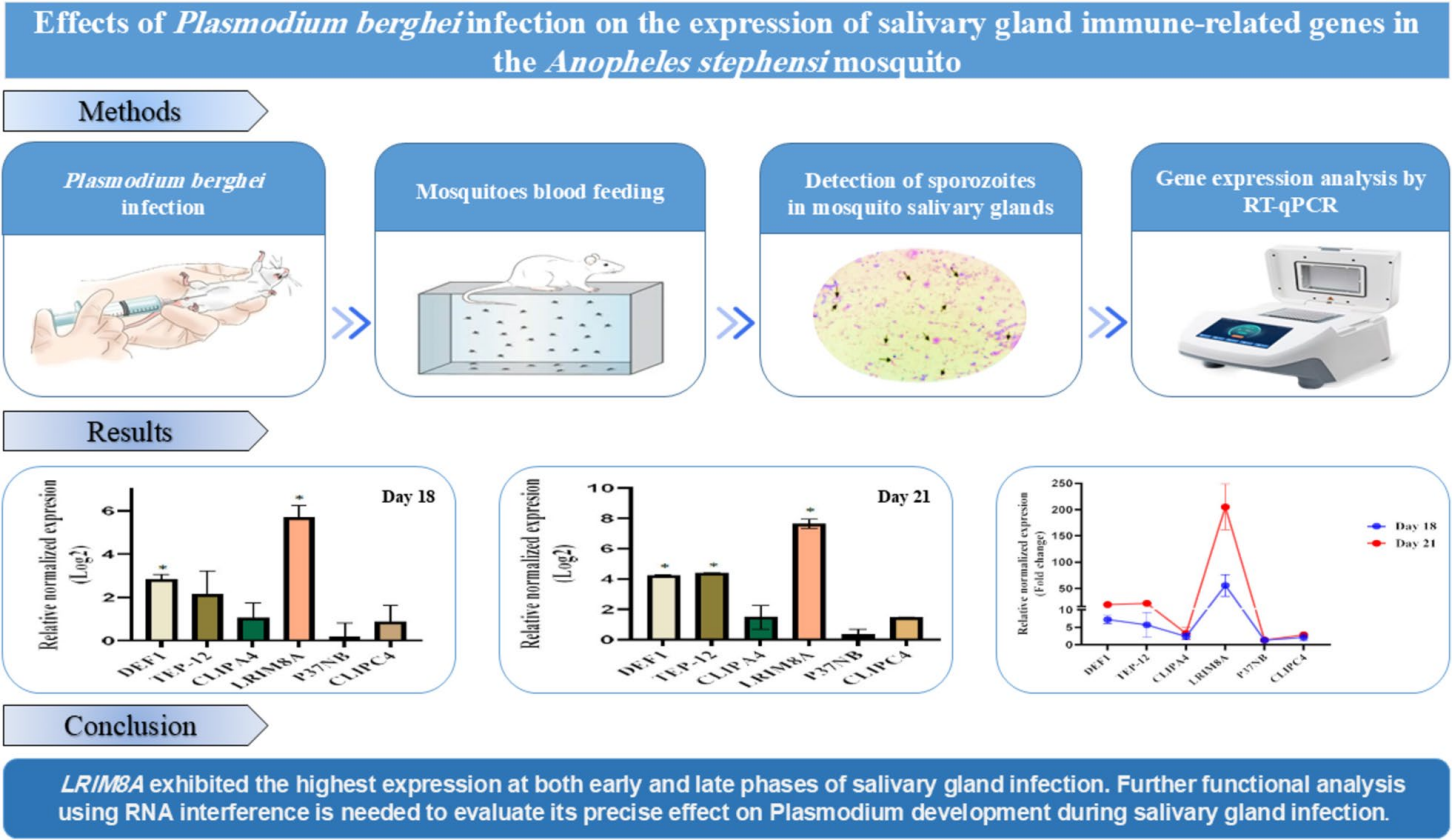

**Supplementary Information:**

The online version contains supplementary material available at 10.1186/s12936-025-05430-6.

## Background

Malaria is a widespread infectious disease caused by parasites of the *Plasmodium* genus and transmitted through the bite of an infected female *Anopheles* mosquito. According to the latest WHO report, there were 263 million malaria cases globally in 2023, resulting in 597,000 deaths [1]. Recent disruptions caused by COVID-19 have hindered progress in reducing malaria’s global burden. The WHO emphasizes the need for developing optimal tools to enhance malaria control and advance toward elimination in endemic regions [2]. Designing novel transmission-blocking strategies requires a comprehensive understanding of *Plasmodium*-mosquito interactions and the immune responses triggered by the parasite. Gaining deeper insights into these mechanisms will aid in identifying novel targets for malaria transmission-blocking interventions [3].

The life cycle of *Plasmodium* is highly complex, involving multiple developmental stages that sequentially occur in both the *Anopheles* mosquito vector and the vertebrate host [4]. Transmission begins when female mosquitoes ingest gametocytes while feeding on an infected host. Inside the mosquito midgut, male and female gametes fuse to form zygotes, which then differentiate into motile ookinetes that invade the midgut epithelium. The ookinetes subsequently develop into oocysts, which mature and release thousands of sporozoites that migrate to the salivary glands. The transmission cycle is completed when an infected mosquito bites another individual, injecting sporozoites into the host [5]. The mosquito's salivary glands serve as the final site for parasite maturation before transmission to vertebrate hosts, acting as a critical bottleneck in the *Plasmodium* life cycle [6]. The efficiency of malaria transmission largely depends on how effectively the salivary gland immune responses eliminate *Plasmodium* [7]. Despite the crucial role of this organ in parasite transmission, details of its immune mechanisms and their impact on *Plasmodium* infectivity remain limited.

The success of the sexual development of *Plasmodium* depends on its interactions with the *Anopheles* immune system. Therefore, a deep understanding of these molecular interactions is crucial for developing novel transmission-blocking strategies. Unlike vertebrates, *Anopheles* mosquitoes lack an adaptive immune system and instead rely on innate immune responses to combat pathogens. The innate immune system consists of physical and physiological barriers that prevent pathogen entry and control infections. In mosquitoes, the innate immune system has two primary components: cell-mediated and humoral immunity, both of which are essential for pathogen defense. Cellular immune responses involve the phagocytosis and encapsulation of foreign invaders, whereas humoral responses include melanization and the production of antimicrobial peptides (AMPs) [8]. These defense mechanisms are initiated when pattern recognition receptors (PRRs) detect pathogen-associated molecular patterns (PAMPs). Recognition of PAMPs triggers a cascade of reactions, including the activation of serine proteases, ultimately leading to processes such as melanization. Additionally, AMP expression is regulated by intracellular signaling pathways, such as the Toll pathway, whose membrane receptors recognize invading pathogens [9, 10].

Recent advances in high-throughput omics technologies, such as RNA-seq, have enabled the simultaneous analysis of thousands of genes and transcripts, providing deeper insights into host–pathogen interactions and immune responses. Studies have shown transcriptional changes in immune-related genes in *Anopheles* salivary glands following *Plasmodium* infection, highlighting the immune role of this organ [11–13]. Since salivary gland immune responses influence vectorial capacity, a detailed examination of vector-parasite interactions could help identify novel targets for malaria control. To explore potential immune targets, six candidate genes were selected from *Anopheles stephensi* salivary transcriptomic datasets, and expression changes were analysed following *Plasmodium berghei* infection. The *P. berghei* model was chosen due to its established use in mosquito immunity research and the reported similarities in the function of immune-related genes between human and murine malaria models [14, 15].

## Methods

### Candidate genes selection and experimental design

Six candidate genes were selected from salivary gland transcriptomic datasets of *Anopheles stephensi* and *Anopheles gambiae* based on their relevance to key immune pathways and differential expression profiles [11, 12] (Table 1). The selection criteria included: (i) enrichment in immune-related pathways, (ii) significant expression changes (false discovery rate < 0.05) in both mosquito species, and (iii) unknown function in the immune response of *Anopheles* against *Plasmodium*.

**Table 1:** List of selected candidate genes in this study and their immune subclasses

Gene	Immune subclass	VectorBase ID	Species
*LRIM8A*	LRRs	ASTE005918	*Anopheles stephensi*
*P37NB*	LRRs	ASTE005729	*Anopheles stephensi*
*TEP-12*	TEPs	ASTE002262	*Anopheles stephensi*
*CLIPA4*	CLIP	ASTE008188	*Anopheles stephensi*
*CLIPC4*	CLIP	ASTEI11306	*Anopheles stephensi*
*DEF1*	AMP	ASTE011281	*Anopheles stephensi*

*LRIM* Leucine-rich repeat immune molecule, *LRRs* Leucine-rich repeats, *TEPs* Thioester-containing proteins, *CLIP* Clip-domain serine proteases, *DEF* Defensin, *AMP* Antimicrobial peptides

Gene expression analysis was conducted at two time points: an early phase (18 days post-blood meal) and a late phase (21 days post-blood meal), the latter corresponding to the peak presence of *P. berghei* sporozoites in the salivary glands. RT-qPCR was used to compare gene expression in infected mosquitoes and control females fed on non-infected mice.

### Mosquito rearing

*Anopheles stephensi mysorensis* mosquitoes, originally collected from the Chabahar district of Sistan and Baluchistan province, were reared under standard conditions (26–28 ℃, 60–80% humidity, and a 12:12 light/dark cycle) at the National Insectarium of the Pasteur Institute of Iran. The mosquitoes were maintained on a 10% sugar solution. Two experimental groups, a test and a control group, were established, each consisting of 300 adult female mosquitoes aged 3–5 days.

### *Plasmodium berghei* infection

*Plasmodium berghei* parasites (strain ANKA) were maintained by serial passage (3–5 passages) in 4- to 6-week-old female BALB/c mice (14–17 g) following previously described protocols [16, 17]. All animal procedures were approved by the Committee of Animal Ethics at the Pasteur Institute of Iran (IR.PII.AEC.1401.009). Parasitaemia was assessed 2–3 days after parasite passage using thin blood smears examined by light microscopy. To preserve gametocyte infectivity for mosquito transmission, hyper-reticulocytosis was induced three days before parasite passage by administering phenylhydrazinium chloride (Sigma-Aldrich, St. Louis, MO, USA) intraperitoneally (10 mg/mL in phosphate-buffered saline [PBS]) at a dose of 10 mg per 10 g of mouse body weight. Mosquitoes were allowed to feed on anesthetized mice when parasitaemia levels were between 8 and 12%, with 2–5 exflagellations per field (40 × magnification). Female mosquitoes were fed on either *P. berghei*-infected mice (test group) or non-infected healthy mice (control group) for approximately 40–45 min. Before blood feeding, mosquitoes from both groups were starved for 12 h, and non-fed females were subsequently removed. The mosquito groups were maintained at 19–21 ℃ with 70–80% humidity to allow parasite development. To assess sporozoite presence and monitor the progression of salivary gland invasion, dissected glands from the test group were examined microscopically between days 17 and 24 post-blood feeding.

### Detection of *P. berghei* infection by qPCR

Detection of *P. berghei* infection in the test group was conducted using a qPCR approach targeting the *P. berghei* cytochrome *b* (*cytb*) gene (Accession No. DQ414645). For this purpose, 30 mosquitoes were randomly selected from the test group on day 21 post-blood feeding, and their heads and thoraces were dissected. DNA was extracted from each head-thorax tissue sample using the Tissue Genomic DNA Extraction Kit (Parstous, Iran), following the manufacturer’s instructions. A 194 bp fragment of the *cytb* gene was amplified using the primer pairs PbF 5′-TTATGGAGTGGATGGTGTTTTAG-3′ and PbR 5′-TGTCCCCAAGGTAAAACATAAC-3′. PCR reactions were performed in a final volume of 20 μL containing 10 μL of SYBR Green master mix (Parstous, Iran), 500 nM of each primer, and 2 μL (30 ng) of template DNA. The reactions were run on a Rotor-Gene Q System (QIAGEN, Germany) under the following conditions: initial denaturation at 95 °C for 15 min, followed by 40 cycles of 95 ℃ for 10 s, 45 ℃ for 10 s, and 72 ℃ for 10 s. Amplification was followed by a melting curve analysis with a dissociation temperature range of 65–95 ℃ to confirm specific amplification of the target sequence. To determine the assay’s limit of detection, dissected salivary glands from 10 *P. berghei*-infected mosquitoes were used, as described previously [18]. Briefly, the total number of sporozoites in pooled salivary glands was estimated using a haemocytometer. Extracted DNA from the pooled salivary glands was serially diluted tenfold up to 1:1,000,000, and each dilution was subjected to qPCR to determine the Cq threshold limit of detection. To confirm specific amplification of the *P. berghei cytb* gene, a qPCR assay was also performed on DNA extracted from 10 randomly selected mosquitoes from the control group.

### RNA extraction and cDNA synthesis

Mosquitoes from both test and control groups were cold-anesthetized on ice, and their head-thorax tissues were dissected on days 18 and 21 post-blood feeding, then stored at − 80 ℃. RNA was extracted from pooled head-thorax tissues (n = 10) using the Total RNA Extraction Kit (Parstous, Iran). RNA quantity and purity were assessed using a DeNovix DS-11 spectrophotometer (DeNovix Inc., Wilmington, USA), and RNA integrity was confirmed by agarose gel electrophoresis visualized under a UV transilluminator (UVItec, Cambridge, UK). To remove genomic DNA contamination, total RNA samples were treated with DNase I using the DNA-free™ Kit (Ambion, USA). The efficacy of the DNase treatment was verified in each RNA sample using standard PCR targeting the *An. stephensi* ribosomal *RpS7* gene (Accession No. AF539918.2). First-strand cDNA was synthesized from 200 ng of DNase-treated total RNA using a qPCR cDNA synthesis kit (Parstous, Iran), which included a combination of oligo(dT) and random hexamer primers. 

### RT-qPCR assay

Primers were designed using Oligo 7.6 software, and their efficiency was initially assessed through conventional PCR using cDNA obtained from a pool of 10 female mosquitoes maintained exclusively on a sugar diet. Before proceeding with relative quantification, the amplification efficiency of each target gene and the endogenous reference gene (*RpS7*) was verified using standard curve analysis with serial 1:5 dilutions of cDNA. Each qPCR reaction (20 µL total volume) contained 125 nM of each forward and reverse primer, 10 µL of 2 × SYBR Green master mix (Parstous, Iran), and 2 µL of cDNA (5 ng). The PCR conditions were identical for all primers and included an initial denaturation step at 95 ℃ for 15 min, followed by 40 cycles of 95 ℃ for 10 s, annealing at the primer-specific temperature (Table 2) for 10 s, and 72 ℃ for 10 s, with a final melt curve analysis from 65 to 95 ℃. The expression profiles of the candidate genes were assessed at 18 and 21 days post-blood feeding. Each of the three independent biological replicates (with separately prepared total RNA) was analyzed in triplicate for each gene. qPCR results were analysed using Rotor-Gene Q software, and expression levels were normalized to the *RpS7* endogenous reference gene.

**Table 2:** Primers used in RT-qPCR analysis

Gene	Primer name^*^	Sequence (5′ to 3′)	Annealing temp (°C)	Expected size (bp)
*RpS7*	S7 F	ATGGTGTTCGGTTCCAAGGTG	57	108
	S7 R	CTTCAGATCCGAGTTCATTTCCAG		
*LRIM8A*	LRI F	TGGCTGCCTATCCTGGTGTATATTTC	56	148
	LRI R	ATCTCCTTCTCACTTTCCAGCAC		
*P37NB*	P37 F	AGAACAACGCCTTCACGCAC	55	131
	P37 R	TCTCAGCTGAACATTACCGGAC		
*TEP-12*	TEP F	TGAAGACGTTTTCCCGCTGTCC	58	146
	TEP R	TTTGATTGCTTGTAGTGCCTTCCTCC		
*CLIPA4*	CLA F	ACGAGATCAATGTCAACTACAATCCAG	57	157
	CLA R	ATTCCACCAATATTACGAAGTCCACAG		
*CLIPC4*	CLC F	TGGAGAAGGATGTCGTGTTCAAG	57.5	130
	CLC R	TCGGCTCGCATGCCATTTC		
*DEF1*	*DEF1* F	AACACGCTCCTGGATGAACTG	58	155
	*DEF1* R	TATCCGCCACGTAAGCGACGAG		

*RpS7* Ribosomal protein S7, *LRIM* Leucine-rich repeat immune molecule, *TEPs* Thioester-containing proteins, *CLIP* Clip-domain serine proteases, *DEF* Defensin

^*^Forward (F) and reverse (R) primers

### Statistical analysis

Graphs and statistical analyses were generated using GraphPad Prism 9 software. Relative gene expression was calculated using the 2^−ΔΔCq^ method, with values compared to the control group [19]. Data are presented as mean ± SEM. Statistical analysis was performed using an unpaired *t*-test with Welch’s correction. *P* < 0.05 was considered to indicate statistically significant differences.

## Results

### High infection rate of salivary glands

*Plasmodium berghei* infection in the salivary glands of *Anopheles* in the test group was confirmed both microscopically and molecularly. Microscopic examination of infected salivary glands indicated that day 18 post-blood feeding corresponded to an early phase of sporozoite infection, while the maximum number of infected glands was observed on day 21 (Supplementary Figure). For the detection of *P. berghei* infection by qPCR, the total number of sporozoites in the pooled infected salivary glands was first estimated. The average sporozoite count in the pooled salivary glands was 26,000,000, or 2,600,000 sporozoites per mosquito. Subsequently, ten-fold serial dilutions up to 1:1,000,000 of DNA extracted from the pooled salivary glands were used to estimate the Cq threshold limit of detection. Standard curve analysis of serially diluted DNA extracts from pooled salivary glands resulted in an R^2^ value of 0.98 and a slope of −3.38. The Cq threshold limit of detection was 34, beyond which no sporozoite was detected using *cytb* gene amplification. Sporozoites were detected in 100% of analyzed mosquitoes (n = 30) using qPCR for amplification of the *cytb* gene of *P. berghei*. The mean Cq value of the analyzed mosquitoes was 18.84 ± 2.33, confirming high levels of sporozoite infection in the salivary glands of the mosquitoes. To ensure correct target sequence amplification, melt curve analysis resulted in a single peak at 77.03 ± 0.33 ℃ in all of the analysed samples. *P. berghei* sporozoites were not detected in any of the control mosquitoes.

### Gene expression analysis of selected genes during *P. berghei* infection

The gene expression of six selected immune-related genes at different time points (18 and 21 days after blood feeding) was studied in infected mosquitoes in comparison to non-infected control mosquitoes. The time points were chosen to coincide with the early (day 18) and late (day 21) phases of salivary gland invasion by sporozoites. The selected genes belong to different innate immune pathways in *Anopheles*. Two genes (*LRIM8A* and *P37NB*) encode members of the Leucine-rich repeats (LRRs) subclass, and one gene (*TEP-12*) encodes a Thioester-containing protein (TEP). Both subclasses are part of the Pattern-recognition receptors (PRRs) gene family, which encodes proteins that identify specific patterns on pathogen surfaces. Two other genes (*CLIPA4* and *CLIPC4*) encode Clip-domain serine proteases (CLIPs), while the remaining gene (*DEF1*) encodes an antimicrobial peptide (AMP).

### Gene expression changes of selected genes at the early stage of salivary gland infection

**Fig. 1:**
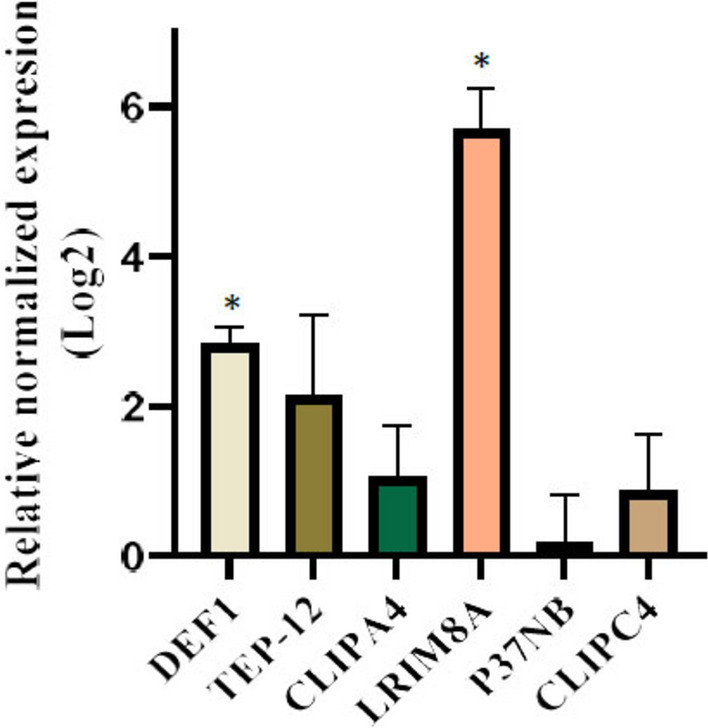
Relative normalized expression of selected immune-related genes during the early stage (day 18) of salivary gland infection by *Plasmodium berghei* sporozoites. Gene expression levels in infected mosquitoes are presented as Log_2_ fold change relative to non-infected (control) mosquitoes, calculated using the 2^−ΔΔCq^ method. RT-qPCR data represent the mean of three biological replicates ± SEM. *P*-values were calculated using an unpaired, two-tailed t-test, and significant differences in gene expression between infected and control groups are indicated by an asterisk

As shown in Fig. 1, shortly after salivary gland infection by sporozoites on day 18, the expression of *LRIM8A* (LRRs subclass) and *DEF1* (AMPs subclass) was significantly higher in infected mosquitoes compared to control mosquitoes fed on non-infected blood (*LRIM8A*: *t*(3.00) = 17.58, *P* < 0.001; *DEF1: t*(1.54) = 10.79, *P* < 0.05). However, the expression levels of the remaining genes (*P37NB*, *TEP-12*, *CLIPA4*, and *CLIPC4*) did not significantly change in the infected salivary glands compared to the non-infected organ (*P* > 0.05). Among all the genes analysed, *LRIM8A* exhibited the highest expression level during the early stage of salivary gland infection.

### Gene expression analysis of selected genes at the late stage of salivary glands infection

**Fig. 2:**
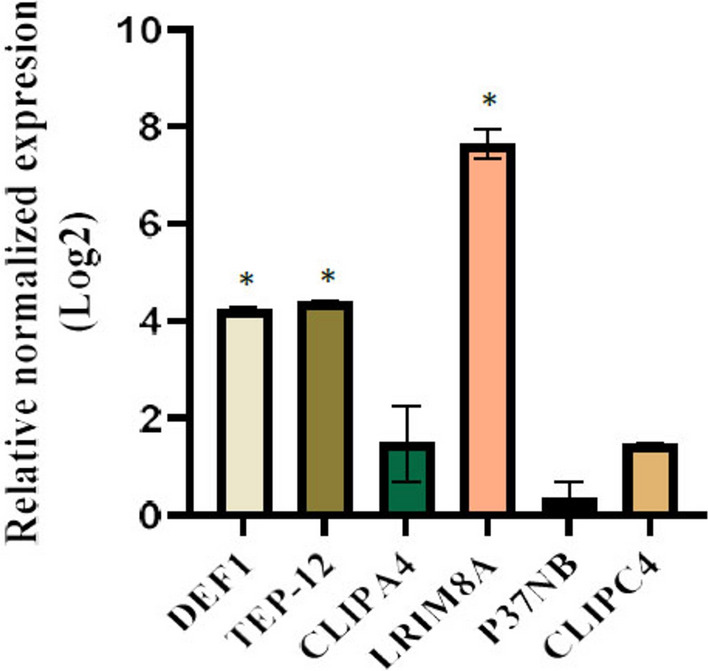
Relative normalized expression of selected immune-related genes during the late stage (day 21) of salivary gland infection by *Plasmodium berghei* sporozoites. Gene expression levels in infected mosquitoes are presented as Log_2_ fold change relative to non-infected (control) mosquitoes, calculated using the 2^−ΔΔCq^ method. RT-qPCR data represent the mean of three biological replicates ± SEM. *P*-values were calculated using an unpaired, two-tailed t-test, and significant differences in gene expression between infected and control groups are indicated by an asterisk

At the late stage of salivary gland infection, when the maximum number of sporozoites resides in the salivary glands, a further increase in the expression of *LRIM8A* and *DEF1* was observed in infected mosquitoes compared to non-infected controls (*LRIM8A*: *t*(3.04) = 42.58, *P* < 0.0001; *DEF1*: *t*(3.06) = 11.30, *P* < 0.05; Fig. 2). Notably, *LRIM8A* showed significantly higher transcript abundance at this stage (*P* < 0.05, Fig. 2). In addition, a significant increase in *TEP-12* transcript levels was detected 21 days after the blood meal (*t*(3.01) = 16.38, *P* < 0.001; Fig. 2). The expression level of *LRIM8A* was higher than that of *DEF1* and *TEP-12* during the late stage of salivary gland infection. Importantly, the expression levels of *P37NB*, *CLIPA4*, and *CLIPC4* in infected mosquitoes remained comparable to those in non-infected controls (*P* > 0.05; Fig. 2).

### Overall variations in the transcriptional abundance of selected genes during the early and late phases of salivary gland infection

**Fig. 3:**
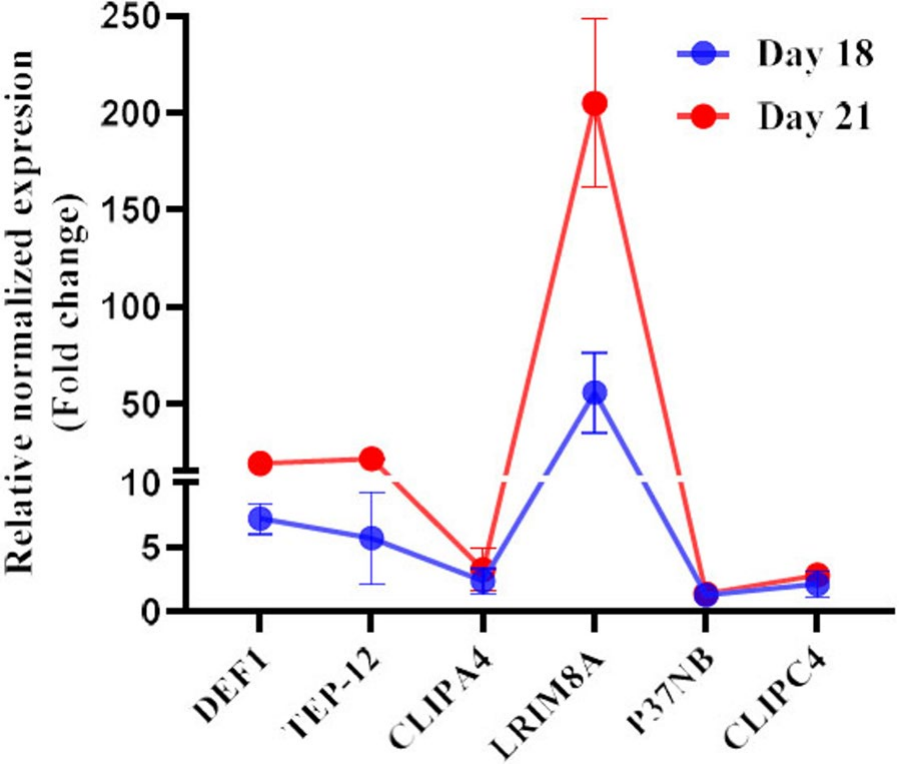
Comparison of transcript variations of selected immune-related genes at early (day 18) and late (day 21) phases of salivary gland infection by *Plasmodium berghei* sporozoites. Gene expression levels are presented as Log₂ fold change relative to non-infected (control) mosquitoes, calculated using the 2^−ΔΔCq^ method. Data represent the mean ± SEM from three biological replicates. Among the analyzed genes, *LRIM8A* exhibited the highest expression level in infected salivary glands at both time points

The transcript variations of selected genes were compared at the early and late phases of salivary gland infection to evaluate the general trends in transcript modulation. As shown in Fig. 3, *DEF1* and *LRIM8A* transcript abundance consistently increased between 18 and 21 days after the blood meal. However, *TEP-12* was only overexpressed during the later phase of salivary gland infection (day 21). Among all the highly expressed genes, *LRIM8A* exhibited the highest expression levels in infected salivary glands during both the early and late phases.

## Discussion

*The Plasmodium* parasites encounter major bottlenecks at two critical stages during its development in mosquitoes: one in the midgut, when migrating ookinetes traverse the midgut epithelium, and the other in the salivary glands, when sporozoites invade this organ [20]. Studies have indicated that *Anopheles* innate immunity contributes to parasite population losses during these developmental bottlenecks [21, 22]. In this study, the expression changes of six immune-related genes were assessed in the salivary glands of *An. stephensi* after *P. berghei* infection. Currently, none of these selected genes have known functions in the immune responses of *An. stephensi*. However, previous clustering studies suggest that these genes are enriched in immune pathways and may play a role in immunity during *Plasmodium* infection [11, 12]. In this investigation, gene expression analysis was carried out at two different time points (days 18 and 21). qPCR analysis indicated that all the analyzed mosquitoes had a high infection rate with sporozoites in their salivary glands. Thus, the observed changes in the expression profiles of the selected genes might be a result of *P. berghei* infection of the salivary glands. The selected genes belong to three important immune pathways in *Anopheles*: the PRRs gene family (*LRIM8A*, *P37NB* and *TEP-12*), the CLIP serine proteases family (*CLIPA4* and *CLIPC4*) and the AMPs (*DEF1*).

When triggered by an invading pathogen, pattern recognition receptors (PRRs) recognize specific patterns of the pathogen and can induce direct defense mechanisms, such as microbial destruction through encapsulation or phagocytosis, as well as indirect mechanisms, including the activation of intracellular signaling pathways that lead to gene expression [23, 24]. In this study, *LRIM8A* expression was upregulated at the early phase of salivary gland infection. Furthermore, the transcript abundance of *LRIM8A* consistently increased during salivary gland infection, with the highest expression observed on day 21, when a large number of sporozoites were present in the organ. Thus, it can be concluded that *LRIM8A* is a key gene with an important role in the immune response against *P. berghei*. This finding is consistent with previous studies highlighting the crucial function of other members of the *LRIM* gene family in immunity against *Plasmodium* [25, 26]. In this study, a significant increase in the transcript abundance of *TEP-12*, a member of the PRRs gene family, was observed at 21 days after the blood meal, suggesting that the increase in parasite load induces *TEP-12* expression. Previous studies have indicated that *LRIM1* and *APL1 C*, two LRR proteins also belonging to the PRRs gene family, form a complex that stabilizes mature *TEP1* [26]. This stabilization is necessary for the destruction of *P. berghei* ookinetes during midgut invasion in *An. gambiae* [26]. Although this study focused on the salivary glands, the results are consistent with previous findings in the midgut, where the PRRs gene family was among the most highly represented subclasses. Overexpression of *LRIM8A* and *TEP-12* during salivary gland infection, as observed in this study, suggests their involvement in the recognition and inhibition of sporozoites.

Antimicrobial cationic peptides play a crucial role in innate defense against invading parasites. The cationic and amphipathic nature of these peptides facilitates their preferential binding to the negatively charged membranes of pathogen cells, leading to their destruction by permeabilizing the cell membranes [27]. The synthesis of antimicrobial peptides (AMPs) is the final step in the humoral immune responses of *Anopheles*, and their expression is induced by various pathways [28]. In this study, the transcript abundance of *DEF1* was found to increase during the early stage of salivary gland infection and continued to rise in the later phase. The overexpression of *DEF1* at both time points suggests its important role in the effector mechanisms against sporozoites. It is known that the overexpression of AMPs in the mosquito midgut and haemolymph can decrease the prevalence of sporozoites and moderately reduce the number of invading sporozoites in the salivary glands of transgenic *An. stephensi* [29]. However, another study indicates that sporozoites are relatively resistant to the cytotoxic activity of AMPs [30]. This phenomenon might result from the parasite's invasion mechanisms against the immune responses of *Anopheles*. Further functional analysis is needed to elucidate the role of *DEF1* overexpression in the ultimate fate of sporozoite transmission.

It is important to note that not all candidate genes were regulated by sporozoite infection in the salivary glands in this study. The absence of expression changes in *P37NB* (a gene from the PRR family), *CLIPA4*, and *CLIPC4* (both belonging to the Clip-domain serine proteases) does not necessarily exclude their potential involvement in immune responses against *Plasmodium*, as post-transcriptional regulation or protein-level modifications may influence their function. Further studies are needed to clarify their possible roles in the immune responses of the salivary glands.

A major limitation of this study is the absence of functional assays, such as RNAi-mediated gene knockdown, which could provide direct evidence of the roles of these genes in *Plasmodium* infection. Future studies should incorporate such approaches to validate the functional relevance of these immune-related genes. Additionally, while *P. berghei* serves as a useful model for studying mosquito immune responses, further validation in *P. falciparum* systems is necessary to confirm the applicability of these findings to human malaria transmission. Despite these limitations, this study provides a foundation for future research into mosquito salivary gland immunity. Identifying key immune effectors in this organ could contribute to novel vector control strategies aimed at reducing malaria transmission.

## Conclusion

This study highlights the dynamic immune responses of *An. stephensi* salivary glands following *P. berghei* infection. Notably, only three genes showed significantly increased expression during the later phase of infection (day 21). The highest expression levels of *LRIM8A* at both early and late phases of salivary gland infection underscore its potential as a key immune effector. While further functional validation is required, this study advances the understanding of mosquito immune mechanisms and provides insights for developing novel malaria control strategies.

## Supplementary Information

Additional file 1.

## Data Availability

No datasets were generated or analysed during the current study.
