# Supplementary material for: Effects of Plasmodium berghei infection on the expression of salivary gland immune-related genes in the Anopheles stephensi mosquito
Source: Malar J. 2025 Jun 6;24:180. doi: 10.1186/s12936-025-05430-6 (PMC12144816; doi:10.1186/s12936-025-05430-6)
Supplement: Supplementary file 1 — Additional file 1. [file 12936_2025_5430_MOESM1_ESM.pdf]

A B

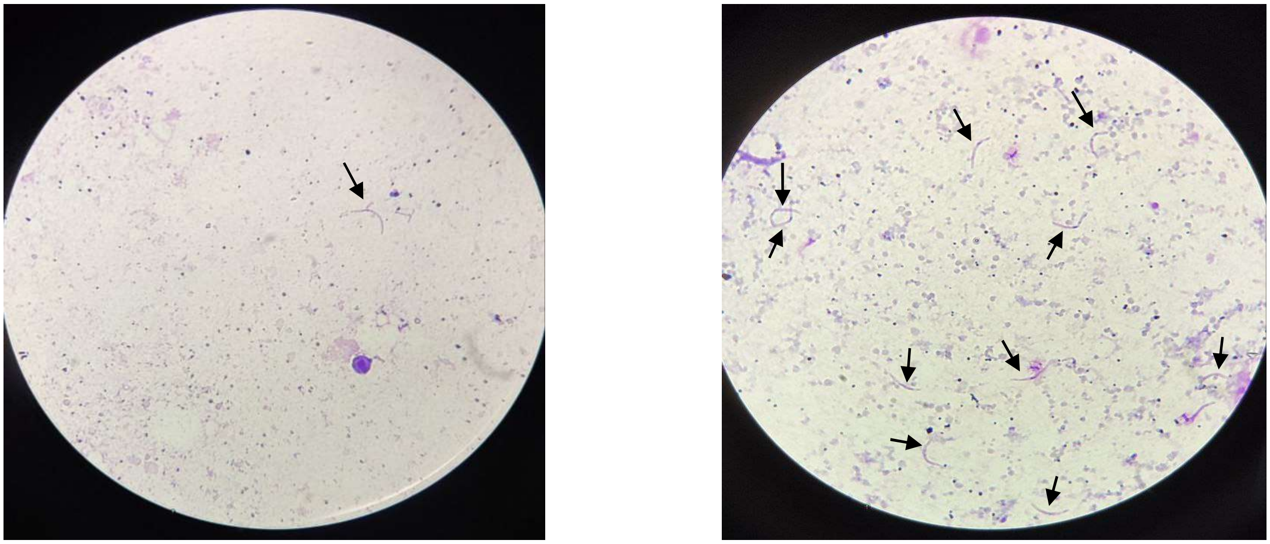

Supplementary Figure. Microscopic images (100× magnification) of dissected *Anopheles* salivary glands after Giemsa staining. Microscopic examination of infected salivary glands revealed that day 18 post-blood feeding corresponded to the early phase of sporozoite infection (A), while the highest number of infected glands was observed on day 21 (B). Arrows indicate the location of sporozoites.
